# A Plea for High Potencies

**Published:** 1882-05

**Authors:** Charles Julius Hempel


### CLINICAL BUREAU.

### A PLEA FOR HIGH POTENCIES.

By the Late Charles Julius Hempel, M. D.

In the "Advertisement of the Editors" of the *Homœopathic Examiner*, 1845, Dr. Hempel writes as follows: "As regards the different potencies, I strictly follow the example of our master. Hahnemann recommends the high potencies as those that *will act most promptly and safely;* my own experience has never, *not in one instance*, contradicted that statement. Even from the 30th potency of *Coffea* I have seen beneficial effects in an affection similar to Purpura hæmorrhagica, although Hahnemann here recommends the lower preparations of that drug. I shall not hesitate to use the 200th and even the 2000th potency of some drugs, as soon as they shall be in my possession. Every homœopathic physician has had cases in his practice where a certain drug was indicated, and yet where it did not act. In all such cases I recommend the use of the highest potencies. In the next number of our journal I shall report a few cases of cure by the highest potencies, where no other potency of the same drug would act."

The following case was reported by Dr. Hempel (page 289 of *Homœopathic Examiner*):

### A Case of Chronic Ovaritis and Leucorrhœa, Successfully Treated by One Dose of Platina 200.

A most beautiful instance of the action of the highest potencies has lately occurred in our practice. We deem it absolutely necessary to record all those cases which furnish a striking illustration of the truth of our law, and of the efficacy of our remedies. Successful cures are the only argument which we now possess in favor of our potentized remedies. There is nothing in known science by means of which we can account for their efficacy, especially for the efficacy of the highest potencies. We will now record a case of cure by the 200th potency of *Platina*, which will be found to be beyond all cavil. As regard the correctness of our statement we are willing to give every guarantee, and we pledge our word that not a single line has been added to the original record.

*Platina* is the remedy by means of which this almost miraculous cure has been effected. Of the great use of *Platina* in affections of the uterine system we have long become convinced by most striking cures. Not long ago, we were called to a lady, who had been under allopathic treatment for six successive months for the following symptoms: Last year she was suddenly attacked with metrorrhagia and rigor of the right arm, globus hystericus, violent pressure upon the chest and a compressive pain in both temples, which was so violent that her family assured us she would attempt her life if she were not prevented by force. The metrorrhagia occurred at every monthly period, all the other symptoms, especially the headache and the globus hystericus, remained permanent complaints, and, if possible, were worse at the time of the menses. A few doses of *Platina* removed the symptoms entirely. In the case of another lady, who was affected with spinal irritation, *Platina* relieved, as by a charm, a dreadful itching in the uterus. This itching had lasted for several days and nights without interruption, and became so excessive at one time that the lady sent for us in the middle of the night, requesting to be relieved. One pellet of *Platina* 1000 quieted her, as by a charm, and the itching never returned.*

The subject of the present case is a young lady of twenty years. At the age of twelve she was attacked with leucorrhœa, which continued ever since, up to the time we were requested to attend her. This discharge was uninterrupted day and night; in taking down a record of her symptoms we took especial pains to inquire into that point with great minuteness; this leucorrhœa was like the white of an egg, and otherwise inoffensive, but debilitating. At the age of fourteen the patient began to menstruate, the first catamenial discharges being without any pain. At the age of fifteen the patient was seized with violent dragging, tearing pain and soreness in the region of the left ovary, the dragging extending through to the small of the back. This pain was especially violent at the time of

* In another case of furious itching in the region of the mons veneris, of six years' standing, and which sometimes was so excessive that the lady when walking in the street, had to enter an alley or some place where no one noticed her, and where she might relieve herself by violent scratching, one dose of *Kal. carb.* 30 effected a perfect and permanent cure. The itching was very deep-seated.

her menses and otherwise as permanent as the leucorrhœal discharge.

Simultaneously with the occurrence of that pain the catamenia assumed a morbid character; the blood had a blackish appearance, it came in clots, and the flow lasted from eight to ten days. At the same time the patient was seized with a violent compressive pain in the temples, and a sense of weight upon the chest, as if it would be crushed by a mass of lead; these symptoms remained likewise permanent complaints. The soreness in the region of the ovary gradually extended over the whole left side, and was especially violent in the small of the back, where it extended across either side of the spine. Two or three times every day the patient experienced violent shootings from the side along the lower portion of the left mamma. These shootings were excessively painful, and accompanied with nausea and giddiness; the patient thought that the nausea and giddiness were caused by the pain. For the last three years the patient had not been able to rest on the left side, partly on account of the soreness, and partly on account of those shootings, which came on as soon as she attempted to lie on that side. For the last three years the pain in the region of the left ovary had changed to a dull, heavy, gnawing pain, and the soreness in the side had greatly augmented. The patient's strength had been failing for some years past; she felt languid in the morning, when rising from her bed she felt weary and broken down, her eyes had retreated into their sockets and were surrounded with blue circles; her spirits were drooping; she avoided society, or rather had an aversion to it; she was extremely melancholy, and, as she had never improved a hair's breadth under the treatment of the best allopathic physicians, her parents began to feel concerned about her.

The symptoms pointed so evidently to *Platina*, that we prescribed it off-hand, dissolving two pellets of the 200th potency in half a tumblerful of water, and requesting the patient to take a teaspoonful night and morning. Eight days after the first visit we saw the patient again, and received from her the following statement: For the first three days that she took the medicine every symptom was worse, and she expressed her firm belief that this aggravation of her sufferings must have been owing to the medicine, for she had never felt such a remarkable exacerbation of pain at any time previous; and during the period of this exacerbation she had been surrounded with remarkably pleasant and cheering influences, which she thought would have relieved her sufferings, if the medicine had not prevented it. She took in all six doses of the medicine. On the morning of the fourth day she felt better than she had ever done before; and on the eighth day, when we saw her again, every one of her symptoms had entirely disappeared, with the exception of an occasional discharge from the vagina, which however was very slight, and nothing but a little watery mucus. She took *Platina* 30 in water, and after the first dose the leucorrhœa stopped entirely, and, up to this moment, during a space of three weeks the patient's health has remained perfect. She says that she feels as if a new life had come into her. We ought to state, upon positive information, that the young lady has not in the least been exposed to influences which might have been instrumental in removing her trouble. Besides she has declared emphatically that she knows she has been cured by the medicine. By turning to the materia medica, we shall find the whole group of symptoms of our case confirmed as a *Platina* disease. Let us examine:

*Moral symptoms:* Scarcely any remedy could be more suitable to the moral symptoms of our patient than *Platina*, as may be seen from the first 27 symptoms.

*Head:* The patient complained of compressive cramp-pain in the temples; see symptoms 53 to 62.

*Chest:* Weight on the chest; see symptom 308.

*Mamma:* Shooting along the mamma; see symptoms 319 and 321.

*Ovary:* Dragging pain; see symptom 286, the correspondence is very remarkable.

*Left side of the back:* Soreness; see symptom 331.

*Leucorrhœa:* Albuminous; see symptom 299.

*Menstrual discharge:* Profuse; see symptoms 292 to 297.

As we said above the cure in this case is complete, so far at least, and by the 200th potency of *Platina*. Even if any of the symptoms should reappear hereafter, we feel confident that *Platina* will control them at once. The cure, so far, cannot be denied, no matter how you twist it and turn it. That the administration of two pellets of the 200th potency of *Platina* should be followed by such results as we have witnessed in the present case, is a mystery to us, and truly marvelous; but it is a fact, a stubborn, incontrovertible fact, and facts like these should induce the systematic disbelievers in the power of the high potencies to forsake all unfounded opposition, and to examine into the facts and principles of our arts with minds that are open to truth and testimony, no matter in what shape and from what side it comes.

